# Why the COVID-19 Pandemic Could Increase the Corporate Saving Trend in the Long Run

**DOI:** 10.1007/s10272-021-0949-x

**Published:** 2021-01-26

**Authors:** Markus Demary, Stefan Hasenclever, Michael Hüther

**Affiliations:** 1 Geldpolitik und Finanzmarktökonomik, German Economic Institute, Konrad-Adenauer-Ufer 21, 50668 Köln, Germany; 2 German Economic Institute, Konrad-Adenauer-Ufer 21, 50668 Köln, Germany

## Abstract

Given the global trend in corporate saving over the last decades, the COVID-19 crisis raises doubts about the persistence of companies’ saving behaviour due to the losses which have occurred in many companies caused by the isolation of households and by lockdowns. Before the pandemic, corporate net lending activities had been increasing for decades due to various factors ranging from the rise in uncertainty after the global financial crisis to the increased reliance on internal funding for research and development expenditures. In Germany, the rise in corporate saving was accompanied by an increase in equity capital and a reduction in the corporate sector’s reliance on bank loans. This article argues that the coronavirus crisis is most likely to interrupt the trend in corporate saving in the short run due to the decline in companies’ revenues. Nonetheless, similar to the pattern observed in the aftermath of the financial crisis, it seems reasonable to conjecture that the COVID-19 shock will strengthen corporate saving in the long run as companies may attempt to restore their liquidity and equity capital buffers to better prepare for future shocks. This will in turn create downward pressure on real interest rates and complicate the conduct of monetary policy.

The debate about the low interest rate environment embraces an excess of saving over investment, i.e. too many funds in search of investment projects. One strand of the research literature focuses on demographics and ageing, which increases the need for households to accumulate financial assets (Krueger and Ludwig, 2007; von Weizsäcker, 2014). Another strand of the literature focuses on the trend of rising corporate saving, which took place in most countries before the COVID-19 crisis, including the ten largest economies, and which was pervasive across industries (see Demary et al., 2020).

**Markus Demary**, German Economic Institute (IW), Cologne, Germany.

**Stefan Hasenclever**, German Economic Institute (IW), Cologne, Germany.

**Michael Hüther**, German Economic Institute (IW), Cologne, Germany.

While the coronavirus pandemic has led to a rise in household saving in the short term due to isolation and increasing uncertainty about future employment and income, the saving rate of the corporate sector has turned negative. This raises the question of how COVID-19 affects the trend in corporate saving in the long term.[1]

## What is corporate saving?

From an individual household's perspective, saving is the part of the after-tax income that is not spent on consumption. If the household's saving is positive, the household will accumulate assets, i.e. its net wealth will increase or its debt will decrease. If the household wants to consume more than its after-tax income, additional funds have to be borrowed or the household's stock of wealth has to be reduced by selling assets, i.e. its net wealth decreases.

From a flow-of-funds perspective, the term *saving* is similarly defined for companies, though the distinction between *gross saving* and net lending becomes relevant. Gross saving is defined as gross profits less dividends, i.e. retained profits from a company's balance sheet perspective. Gross saving can either be used to finance investment expenditures or allocated to improve the company's net financial position by either accumulating assets

1 We analyse this in Demary et al. (2020) with a focus on Germany.

Figure 1
**Net lending, cash holding and cash ratio of German companies**

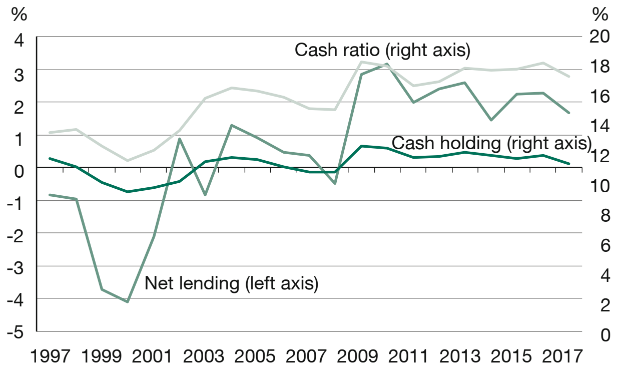

Notes: Net lending in percent of GDP, cash holding in percent of financial assets, cash ratio in percent of short-term financial liabilities.

Sources: Deutsche Bundesbank; Eurostat; own calculations.

or decreasing liabilities. When a company's investment expenditures exceed its gross saving, the company has to attract funding from banks or investors and becomes a *net borrower*. Similarly, when the difference between a company's gross saving and its investment is positive, which is also called *excess saving*, the firm becomes a net lender to the rest of the economy. Altogether, to be a gross saver, the company has to (i) be profitable and (ii) distribute less than 100% of its profits to its shareholders. When a company becomes a *net lender*, its excess saving is allocated either to the purchase of financial assets or to the reduction of liabilities.

Corporate net lending is therefore predominantly allocated in liquid financial assets such as bank accounts or liquid bonds. Thus, the correlation between the business sector's net lending and the business sector's cash holdings is high (see Figure 1). Dao and Maggi (2018) emphasise that this correlation is particularly high for German companies, reflected by a correlation coefficient of 0.84. Aside from cash accumulation, the Deutsche Bundesbank (2019) provides evidence that German corporations also tend to use their excess saving for debt reductions. Accordingly, the amount of cash divided by short-term liabilities (cash ratio) has been rising over time. The cash ratio also highly correlates with the corporate excess saving with a correlation coefficient of 0.92.

In principal, net lending can also be used for equity buybacks. However, evidence provided by Chen et al. (2018) and Dao and Maggi (2018) shows that equity buybacks can only explain a negligible part of the rise in corporate net lending, except in US firms. Research by the Deutsche Bundesbank (2019) supports this finding for the use of corporate saving in Germany.

## What are the drivers of corporate saving?

Traditionally, the business sector has been a net borrower, i.e. companies normally borrow funds to finance their investments in physical assets. The transformation of the corporate sector from being a net borrower to becoming a net lender in the last decades has been raising questions about the causes and implications of this trend.

For analysing the effects of the COVID-19 crisis on corporate saving, this section provides an overview of factors that have driven the trend in the first place. Subsequently, an analysis of the composition of Germany's aggregated corporate sector balance sheet over time follows together with an analysis of the aforementioned drivers that are relevant for the rise in corporate saving in Germany. Since corporate net lending is mostly held in cash and other highly liquid assets as discussed above, a considerable part of the corporate finance literature uses the terms corporate excess saving and liquidity accumulation as synonyms.

### Main drivers defined by the literature

*The precautionary motive of saving*: Dao and Maggi (2018) and Bates et al. (2009) show that companies facing higher idiosyncratic uncertainty accumulate more liquidity to have a buffer against adverse shocks. In addition, Dao and Maggi (2018) illustrate that aggregate uncertainty, proxied by the economic policy uncertainty index (EPU), drives firms' cash hoarding.

*The transaction motive of saving*: Dao and Maggi (2018) and Bates et al. (2009) also find evidence that larger firms tend to accumulate less saving than smaller companies, because large firms face economies of scale when transforming nonfinancial assets into cash, which in turn reduces the need for liquidity buffers.

*The composition effect*: From another point of view, Begenau and Palazzo (2016), Booth and Zhou (2013) and Graham and Leary (2017) show that the rise in corporate saving reflects changes in the composition of companies in the market. Since the 1980s and 1990s, more high-tech and research and development (R&D)-intensive firms – that have been more willing to hold cash compared to incumbent companies – have entered the US stock market. However, Dao and Maggi (2018) show that the wave of R&D-intensive firms only lasted until the early 2000s and has subsequently reversed. In accordance, Chen et al. (2017) also emphasise the pervasiveness of the rise in corporate saving across industries.

Figure 2

**Physical assets, intangible assets and acquisitions of German companies**

in percent of total assets

Source: Deutsche Bundesbank; own calculations.

Figure 3

**Equity capital and bank loans of German companies**

in percent of total assets

Source: Deutsche Bundesbank; own calculations.

*The rising importance of intangible assets*: Falato et al. (2013) argue that the ability of pledging intangibles as collateral for external funding is constrained. Consequently, companies need to accumulate liquidity to have enough internal resources to finance future investments. Supporting this argument, Bates et al. (2009), Pinkowitz (2015) and Dao and Maggi (2018) find evidence that firms with higher R&D spending tend to hold more cash.

*Heightened product market competition*: Della Seta (2011), Morellec et al. (2014) and Lyandres and Palazzo (2016) emphasise that given financial market restrictions, enhanced competition increases firms' reliance on internal funds to finance investment necessary for the survival of the company.

*A rise in foreign income*: Chen et al. (2017) underline that this relationship does not reflect lower tax payments of multinationals realised by profit shifting to tax havens. Instead, they argue that this finding expresses the larger profitability of export-orientated firms. Foley et al. (2007) argue, however, that US multinationals' saving activities can partly be explained by efforts to avoid tax costs associated with the repatriation of foreign income.

*Agency motives*: Jensen's (1986) work implies that firms with entrenched managers and no good investment opportunities are reluctant to pay out dividends. The results of Dittmar et al. (2003), Kalcheva and Lins (2007), Pinkowitz et al. (2006) and Dao and Maggi (2018) confirm the importance of agency problems for cash holding.

## Composition of the corporate sector balance sheet

Accompanied by the rise in net lending over the last two decades, the composition of investment and funding has changed in the German business sector. Physical assets have gradually declined from 30.7% of total assets in 1997 to 22.5% in 2017 (see Figure 2), which is in line with the findings of studies covering the US (e.g. Falato et al., 2013). The decline in physical assets in percent of total assets could be due to heightened importance of intangible goods, which may be associated with larger cash holdings as discussed before. It seems unlikely, however, that German companies have increased their saving to provide funding for future expenditures on intangibles. First, the share of intangibles on total assets is modest, e.g. 1.5% in 2007. Second, in contrast to the US, it has been declining since the early 2000s for a substantial period.

Nevertheless, German companies may have been holding cash for the realisation of targeted acquisition as they have increased in Germany from 9.9% of total assets in 1997 to 19.4% of total assets in 2017 (see Figure 2). However, results of a study from the Deutsche Bundesbank (2019) provide evidence that acquisitions of foreign firms are not significantly related to the corporate gross saving of German firms. Retained earnings of foreign subsidiaries count as both gross saving and direct investment for the German parent company. On the other hand, targeted acquisitions of domestic companies could have been an important driver for the accumulation of liquidity.

On the liability side of the German corporate sector's balance sheet, Figure 3 shows an increase in the equity capital from 20.4% of total assets in 1997 to 31.2% in 2017 (equity capital ratio). Since the issuance of new capital has remained roughly stable over time, we conclude that the corporate sector's excess saving has been mainly used to improve the equity capital position. In this vein, bank loans have been declining from 19.3% of total assets in 1997 to 10.5% in 2017 despite the low interest rate environment. While the reduction in bank loans probably reflects the deleveraging associated with the rise in corporate saving in Germany, it may also review the substitution of bank loans by inter-company loans.

### Relevant factors for the rise in corporate saving

Given the developments on the balance sheets of companies, additional factors have to be taken into account:

First, before the tax reform of 2000, retained profits were taxed at a higher rate than distributed profits, which provided companies with an incentive to distribute profits and thus to operate with lower equity capital ratios. With the reform, tax rates were harmonised to an equal rate, which increased the incentive for companies to retain profits and increase their equity capital ratios. The Deutsche Bundesbank (2019) finds no evidence, however, that the harmonisation of the tax rates affected companies' retained earnings significantly (tax motive).

Second, after the two sharp recessions in 2001 and 2008, companies probably noticed that having a stronger balance sheet made them more resilient to economic shocks. Therefore, they may have been raising their equity capital buffers as well as their liquidity buffers, i.e. they became gross savers as well as net lenders. Additionally, the heightened uncertainty associated with these recessions might have increased firms' willingness to hold cash, i.e. the precautionary motive of saving.

Third, since the 2004 bank regulation known as Basel II, access to credit has been more restrictive for companies with a higher default probability. Because of that, companies have an incentive to increase their equity capital ratios for improving their creditworthiness.

## What is the impact of the COVID-19 pandemic on corporate saving?

To get more insights into the long-term effects of the pandemic on corporate saving activities, we first have to analyse the immediate effects of the COVID-19 shock on firms' balance sheets:

- a decline in gross saving since profits have declined or turned into losses, mainly due to companies in the sectors most affected by the pandemic

- a decline in the liquidity buffers of firms since firms have to continue payments to employees, debtors and suppliers, while revenues have declined

- decreasing equity capital buffers when losses have occurred and/or due to the decline in liquidity

- increasing short-term debt to cover costs in times of deteriorating revenues

- declining net lending or rising net borrowing since financial assets have been reduced and financial liabilities have been increased.

In the short term, the COVID-19 shock may have mitigated corporate saving activities due to a decline in revenues and profits. However, many governments have responded quickly and appropriately by using instruments to secure liquidity. These instruments, e.g. short-time working allowance, tax deferrals, loan guarantees and grants, are aimed at bridging the escalation phase of the crisis to enable the companies to continue their businesses during and after the crisis without serious loss of liquidity and equity. Even if there will not be an enormous wave of insolvencies, which would put the banking system under considerable pressure (Demary and Hüther, 2020), sizeable consequences will continue to have an impact on companies' capital adequacy.

With respect to the long-term effects of the pandemic on corporate saving behaviour, we assume that the precautionary saving motive will have a strong effect on corporate saving in the future, since the other saving motives are less connected to the COVID-19 crisis and more related to longer-term developments and framework conditions. In this vein, we expect companies to restore their liquidity and equity capital buffers in the aftermath of the COVID-19 crisis due to the following most probable developments:

- In the medium term, the economy recovers and revenues of companies will normalise during the recovery, which will lead to rising profits. In addition, many firms may have realised that the build-up of liquidity buffers in the past has strengthened their financial position and made them more robust against adverse shocks. That is why firms might have an incentive to build up their liquidity buffers to be prepared for the next recession, perhaps even beyond the pre-COVID-19 crisis level.

- When banks suffer from credit losses during the recession, they will probably become more restrictive in their lending. This will particularly affect companies with low equity capital buffers. Thus, companies will most likely aim to restore their equity capital buffers to secure their access to external funding.

- Companies will have to repay the short-term debt they accrued during the pandemic before they can apply for new loans to purchase new investment goods, which will lead to low investment accompanying the build-up of liquidity and equity capital buffers.

Given these scenarios, firms will probably have to strengthen their balance sheets in the longer term before they can engage in new investment activities. The periods after the COVID-19 shock will therefore most likely be characterised by a deleveraging process of companies, i.e. a period of low investment and increasing profits, which will translate into rising corporate saving.

## Conclusion

Altogether, the coronavirus crisis seems to have dampened the trend of corporate saving in the short run. In the long run, however, the coronavirus shock will most likely strengthen corporate saving activities, since companies have experienced that having higher equity capital and liquidity buffers can be beneficial in crises in which liquidity dries up. This precautionary saving motive might be reinforced as we expect that the perception of risk is likely to continue to rise in general, as it has after the other severe crises since the turn of the millennium, e.g. the New Economy bubble, 11 September, the Iraq war, the global financial crisis and the European sovereign debt crisis. Hence, from a macroeconomic point of view, the decline in equilibrium real interest rates could persist in light of the corporate saving activities, which will likely be reinforced in the long run as a response to the COVID-19 shock.
